# “Unkinking” the “Kink” Normalizes the Doppler Pattern

**DOI:** 10.3390/diagnostics14141550

**Published:** 2024-07-18

**Authors:** Elaina A. Blickenstaff, Michael O’Shea, Timothy Barry, Reza Arsanjani, John P. Fasolino, Donald J. Hagler, Francois Marcotte, David S. Majdalany

**Affiliations:** 1 Department of Biology, Marshall University, 1 John Marshall Drive, Huntington, WV 25755, USA; 2 Department of Internal Medicine, Mayo Clinic, 13400 East Shea Boulevard, Scottsdale, AZ 85259, USA; oshea.michael@mayo.edu (M.O.);; 3 Department of Cardiovascular Medicine, Mayo Clinic, 13400 East Shea Boulevard, Scottsdale, AZ 85259, USA

**Keywords:** coarctation of aorta, stent, echocardiography, Doppler pattern

## Abstract

Coarctation of the aorta (CoA) comprises 5–7% of congenital heart disease and can present as an isolated narrowing in the aortic arch just distal to the left subclavian artery or can be associated with cardiac abnormalities such as a bicuspid aortic valve, aortopathy, or ventricular septal defects. With the advances in the medical field, intervention on CoA can either be via surgical repair or endovascular stenting. Echocardiography is the mainstay in diagnosing CoA, with tomographic imaging such as magnetic resonance imaging (MRI) or computed tomography providing supplementary assessment of the aorta, valves, and collateral vessels. We present a case of a young hypertensive male who was noted to have a continuous cardiac murmur with diagnostic Doppler pattern of CoA on echocardiography that normalized soon after percutaneous stenting.

A 19-year-old hypertensive male presented to the clinic for the evaluation of a continuous cardiac murmur noted posteriorly. He had a 12 mmHg blood pressure gradient between his right upper and right lower extremities. Upon physical examination, his femoral pulses had a reduced amplitude. On echocardiography, he was found to have normal biventricular size and systolic function, a trileaflet aortic valve, and normal dimensions of the ascending aorta. Suprasternal echo images revealed turbulent flow in the narrowed aortic arch on color flow (white arrow Figure 1A), with increased velocity on continuous-wave Doppler with a “saw-tooth” pattern and antegrade flow in diastole (Figure 1B), with peak and mean gradients of 49 mmHg and 22 mmHg, respectively. In the subcostal view, interrogation of the abdominal aorta revealed a dampened low-velocity signal with continuation of flow through diastole (Figure 1C) indicating coarctation of the aorta. Cardiac magnetic resonance imaging confirmed severe coarctation of the aorta with minimal diameter of 8 mm (white arrow Figure 1D), two centimeters away from left subclavian artery, and extensive collateral vessels (red arrow Figure 1D). The patient was referred for cardiac catheterization, revealing a peak–peak gradient of 24 mmHg across the coarctation which was 2 cm distal to the left subclavian artery. He underwent subsequent stenting with two covered endovascular stents in tandem, which were dilated to a maximum dimension of 20 mm with no residual gradient noted (Figure 1E). The next day, post-procedure suprasternal echo imaging revealed a normalized Doppler flow pattern in the aortic arch with lower flow velocity and no antegrade flow in diastole (Figure 1F). Subcostal window imaging of the abdominal aorta revealed a higher velocity with brisk upstroke and downstroke pulsed-wave Doppler pattern without continuation of flow in diastole and with early flow diastolic reversal (red arrow Figure 1G), representing normal aortic recoil in the setting of no obstruction.

The patient was asymptomatic at the six-month follow-up after the procedure, with blood pressure readings in the high normal range at rest and no murmur noted on examination. He will be completing an exercise stress test in the future to gauge for possible hypertensive response and need to initiate anti-hypertensive therapy. With the increased frequency of intracranial aneurysms in the setting of CoA, the patient had brain imaging performed, revealing no berry aneurysms. Given the long-term sequelae of CoA, which can include arterial hypertension and recurring CoA, the patient will have yearly follow-up in the congenital cardiac clinic with serial cardiac imaging, including echocardiography and chest tomographic imaging, to assess for aortopathy or re-coarctation [1,2].

## Figures and Tables

**Figure 1:**
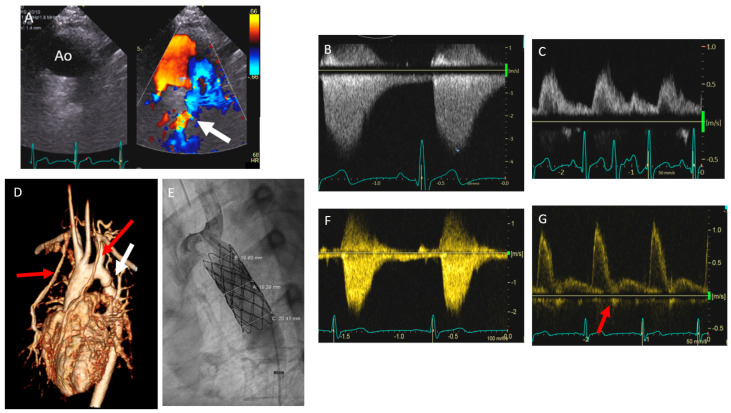
A 19-year-old hypertensive male was noted to have a posterior continuous murmur on cardiac auscultation. Panel (**A**) represents suprasternal echocardiographic imaging of the aortic arch (Ao) revealing turbulent flow in the distal narrowed arch (white arrow) with increased velocities noted on continuous-wave Doppler interrogation (panel (**B**)) with peak and mean gradients of 49 mmHg and 22 mmHg, respectively, and forward flow noted during diastole. Panel (**C**) reveals subcostal echocardiographic pulsed-wave Doppler interrogation of the abdominal aorta revealing dampened low-velocity signal with continuous flow through diastole and absence of early diastolic flow reversal (EDR) suggesting significant coarctation. Panel (**D**) reveals cardiac magnetic resonance imaging confirming severe coarctation of the aorta (white arrow) with extensive collateral vessels (red arrows). Panel (**E**) reveals the descending thoracic aorta post-stenting and angiographic relief of obstruction. Panel (**F**) reveals post-stenting suprasternal echocardiographic continuous-wave Doppler interrogation of the aorta with normalized lower velocity flow pattern without antegrade flow in diastole. Panel (**G**) reveals post-stenting subcostal echocardiographic pulsed-wave Doppler interrogation of the abdominal aorta revealing normal Doppler flow pattern with brisk upstroke and downstroke and presence of EDR (red arrow), suggesting no significant obstruction.

## Data Availability

No new data were created or analyzed in this study. Data sharing is not applicable to this article.

## Abbreviations

CoA: coarctation of the aorta; MRI: magnetic resonance imaging.
